# Is CXCR4 Theranostics in Oncologic, Cardiovascular, and Inflammatory Diseases Really Happening?

**DOI:** 10.2967/jnumed.125.271153

**Published:** 2026-05

**Authors:** Philipp E. Hartrampf, Rudolf A. Werner, Takahiro Higuchi, Margret Schottelius, Andreas K. Buck

**Affiliations:** 1 Department of Nuclear Medicine, University Hospital Würzburg, Würzburg, Germany;; 2 National Cancer Centre, NCT WERA, University Hospital Würzburg, Würzburg, Germany;; 3 Bavarian Centre for Cancer Research, Erlangen, Germany;; 4 Department of Nuclear Medicine, Ludwig Maximilians University, Munich, Germany;; 5 Comprehensive Heart Failure Centre, University Hospital Würzburg, Würzburg, Germany;; 6 Collaborative Research Centre Cardioimmune Interfaces (SFB 1525), Department of Internal Medicine I, University Hospital Würzburg, Würzburg, Germany;; 7 Translational Radiopharmaceutical Sciences, Lausanne University, Lausanne, Switzerland; and; 8 Agora pôle de recherche sur le cancer, Lausanne, Switzerland

C-X-C motif chemokine receptor 4 (CXCR4) is a transmembrane chemokine receptor involved in various biologic processes, including tumor growth, metastasis, and the homing and retention of hematopoietic stem cells or progenitors in hematopoietic sites (1). Under physiologic conditions, CXCR4 is predominantly expressed on T lymphocytes, B lymphocytes, monocytes, macrophages, neutrophils, and eosinophils as well as in tissues of the immune system, with ubiquitous expression detectable in a variety of organs (2). CXCR4, in conjunction with its natural ligand, the C-X-C motif chemokine 12 (CXCL12, or stromal cell–derived factor 1), is of pivotal importance in regulating the mobilization and homing of stem cells (3). Targeted disruption of this system can be used for stem-cell release, such as a scheduled bone marrow transplantation (2).

The interaction of CXCR4 and CXCL12 also has a role in the promotion of angiogenesis, metastasis, and survival of tumors (2). It has been demonstrated that several CXCR4-positive cancers metastasize to the bone and lymph nodes in the presence of CXCL12. This process can be facilitated by the bone marrow, which can provide a protective environment for tumor cells (4). The retention of acute lymphoid and acute myeloid leukemia cells in bone marrow is also facilitated by the CXCR4/CXCL12 axis (5). CXCR4’s aberrant expression in a wide range of cancers and inflammatory conditions has positioned it as a key target in both diagnostics and therapeutics. Radiopharmaceuticals directed against CXCR4 have emerged as promising tools for molecular imaging (6) and radionuclide therapy (7).

The clinical application of CXCR4 imaging in the diagnosis and management of primary aldosteronism has been previously reported (8). Here, we discuss the future of CXCR4-directed imaging and therapy in hematologic malignancies, solid tumors, and cardiovascular and inflammatory diseases.

## HIGH-PERFORMANCE CXCR4-TARGETED TRACER CONCEPTS ARE AVAILABLE

The increasing recognition of CXCR4 as a highly relevant biomarker in cancer and inflammatory conditions has initiated intense efforts toward the development of appropriate molecular imaging probes (9). To date, only the [^68^Ga]Ga-Pentixafor/[^177^Lu]Lu-/[^90^Y]Y-Pentixather theranostic concept (10,11) is available for clinical application, with a growing body of evidence underscoring its clinical utility and therapeutic impact, as we will discuss.

However, the field is expanding, with an increasing number of alternative tracer concepts advancing through the translational pipeline. Most notably, the [^68^Ga]Ga-Pentixafor/[^177^Lu]Lu-/[^90^Y]Y-Pentixather family has been expanded by second-generation ligands with modified linker structures that confer substantially improved CXCR4 affinities and internalization profiles and greater flexibility toward the implementation of non-DOTA-based radiolabeling strategies (12,13). The SPECT analog [^99m^Tc]Tc-Pentixatec is one of the first compounds to enter the clinical arena (14).

Several other peptide-based ligands have recently demonstrated great promise for potential clinical applicability, including tracers based on the octapeptide scaffold LY2510924 (e.g., [^68^Ga]Ga-BL31 (15), [^18^F]AlF-NOTA-SC (16)), optimized EPI-X4 analogs (17), and disulfide-bridged cyclic pentapeptides (e.g., [^18^F]AlF-NOTA-QHY-04 (18)). Preclinically, [^68^Ga]Ga-BL31 showed superior in vivo imaging characteristics compared with [^68^Ga]Ga-Pentixafor, with increased tumor accumulation and reduced kidney uptake, whereas the first clinical data obtained with [^18^F]AlF-NOTA-QHY-04 indicate imaging contrast and tumor accumulation comparable to that of [^68^Ga]Ga-Pentixafor. Taken together, these findings suggest that, while the [^68^Ga]Ga-Pentixafor/[^177^Lu]Lu-/[^90^Y]Y-Pentixather pair currently represents a highly optimized and clinically validated benchmark in CXCR4-targeted theranostics, the next generation of analogs may yet reveal incremental and perhaps meaningful advantages in imaging performance or pharmacokinetics. Ultimately, their true clinical value will need to be established in head-to-head comparative studies.

## CXCR4 PET CAN IMPROVE DIAGNOSTICS AND TREATMENT PLANNING

Several clinical and translational studies have demonstrated that CXCR4-targeted PET provides more than the in vivo visualization of receptor expression—it redefines disease characterization, improves diagnostic accuracy, and guides therapy selection in both hematologic malignancies and solid tumors.

**FIGURE 1.**
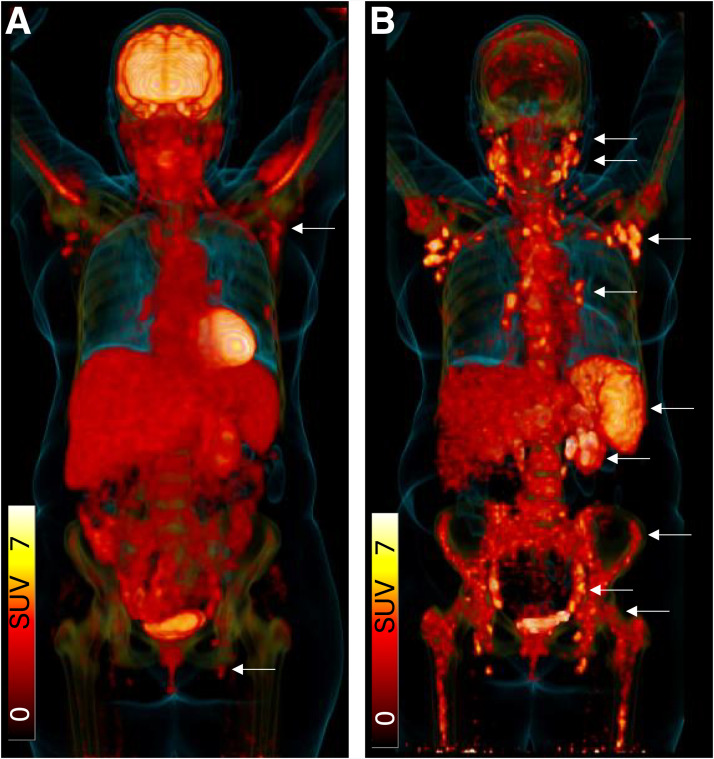
In hematologic neoplasms, CXCR4-directed PET can be more sensitive than [^18^F]FDG PET, particularly in marginal zone lymphoma. PET/CT scans of a 70-y-old woman with initial diagnosis of marginal zone lymphoma are shown, performed within 2 d of each other. (A) [^18^F]FDG PET/CT (3-dimensional view) shows moderately increased tracer uptake in axillary and inguinal lymph nodes (arrows). (B) [^68^Ga]Ga-Pentixafor PET/CT (3-dimensional view) shows additional lesions in cervical, mediastinal, parahepatic, and pelvic lymph nodes as well as bone marrow compartment and spleen, indicating advanced disease.

CXCR4-directed PET has shown potential clinical utility across hematologic cancers. In a multicenter analysis of 690 patients, [^68^Ga]Ga-Pentixafor uptake consistent with disease was observed in roughly 70% of cases (6). The tracer improved staging in 50% of patients with marginal zone lymphoma and altered the therapeutic strategy in one third of patients, underscoring its impact on clinical decision-making (Fig. 1).

Similarly, [^68^Ga]Ga-Pentixafor PET has outperformed conventional [^18^F]FDG PET/CT in mantle cell and marginal zone lymphomas by revealing additional sites of disease and enabling more accurate response assessment (19). Retrospective data have further highlighted its potential use in lymphoma of the central nervous system (20) and a prospective clinical trial in mucosa-associated lymphoid tissue lymphoma (21). These encouraging findings led to the initiation of the prospective phase 3 PTF-301 trial (NCT06125028), comparing [^68^Ga]Ga-Pentixafor with [^18^F]FDG PET/CT in marginal zone lymphoma. Although the study was terminated prematurely in 2025 because of slow recruitment (22), it reflects the strong clinical momentum toward integrating CXCR4 PET into routine diagnostic pathways.

In multiple myeloma and related hematologic malignancies, CXCR4 PET uncovers previously unrecognized lesions in about 20% of patients and complements [^18^F]FDG PET in depicting both intramedullary and extramedullary disease (6). Several phase 1 and 2 studies are prospectively recruiting patients with myeloma (NCT04561492, NCT06871176), B-cell lymphoma (NCT06461182), and other hematologic entities (NCT07122674, NCT05093335, NCT06834412, NCT05255926, NCT06690736) to refine the diagnostic role and therapeutic predictive value of CXCR4 imaging.

Although the strongest evidence for CXCR4 PET lies in hematologic malignancies, experience in solid tumors is growing. [^68^Ga]Ga-Pentixafor PET demonstrates variable diagnostic performance depending on the histologic subtype, with the highest uptake observed in adrenocortical carcinoma, small cell lung cancer (SCLC), and desmoplastic small round cell tumors, and low or negligible uptake in prostate, colorectal, and hepatocellular carcinomas (6,23).

A 2025 systematic review encompassing 831 patients across 26 studies confirmed this variability and underscored that, although overall lesion detectability was lower than that with [^18^F]FDG PET/CT, CXCR4 PET/CT provided superior molecular specificity by directly mapping receptor expression relevant for CXCR4-directed therapy (23). Tumor uptake correlated positively with immunohistochemical CXCR4 expression, confirming biologic specificity, even where absolute signal intensity is modest.

Most recently, a prospective study using [^18^F]AlF-NOTA-QHY-04, a ^18^F-labeled CXCR4-targeting tracer, has expanded the diagnostic horizon for SCLC (18). In 35 patients, this tracer achieved high uptake in both primary and nodal lesions and, crucially, distinguished molecular SCLC subtypes. Uptake was significantly higher in the SCLC-N (NEUROD1-defined) subtype than in other variants and was higher in neuroendocrine compared with nonneuroendocrine subtypes. These findings suggest that CXCR4-directed PET/CT can noninvasively identify aggressive molecular phenotypes, potentially informing prognosis and guiding personalized therapy selection, including immunotherapy in nonneuroendocrine subtypes.

Furthermore, early results in glioblastoma further suggest that CXCR4 signal intensity correlates with World Health Organization grade and recurrence risk, offering a noninvasive imaging biomarker for prognosis and response monitoring (24). Overall, CXCR4 PET is emerging as a complementary diagnostic tool in solid tumors, which is particularly valuable for visualizing receptor biology when [^18^F]FDG uptake is nonspecific or absent, such as in certain neuroendocrine, adrenal, and chemoresistant tumors.

Beyond its diagnostic role, CXCR4 PET serves as a biomarker selection and dosimetry tool for CXCR4-targeted radionuclide therapy. Imaging with [^68^Ga]Ga-Pentixafor reliably identifies patients with high CXCR4-expressing tumor burden suitable for treatment with its therapeutic counterparts, [^177^Lu]Lu- or [^90^Y]Y-Pentixather. This enables individualized activity planning and safety assessment, particularly relevant when used as a conditioning regimen before hematopoietic stem-cell transplantation (25).

Because CXCR4 expression is heterogeneous and dynamically regulated by prior therapy (26–28), PET-based receptor mapping allows adaptive patient stratification and longitudinal response monitoring. The same principle extends to combination regimens involving CXCR4 antagonists or chemokine pathway inhibitors, where imaging can identify responsive molecular subgroups (29).

Overall, CXCR4-targeted PET has evolved from an experimental tracer to a clinical decision-support tool. By refining disease staging, revealing occult lesions, and enabling rational patient selection for CXCR4-directed radiopharmaceutical therapy (RPT) or combination therapy, it bridges diagnostics and therapy in a genuinely theranostic manner. Its strength in hematologic malignancies is already established, and its expanding role in select solid tumors marks the next step toward precision molecular oncology.

## CXCR4-TARGETED RPT SHOWS CLINICAL PROMISE

**FIGURE 2.**
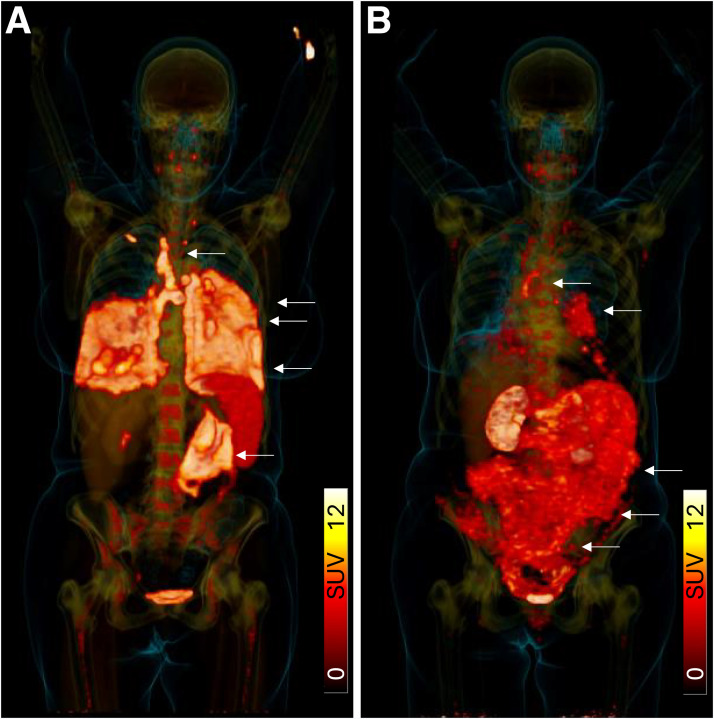
CXCR4-directed RPT with [^90^Y]Y-Pentixather holds promise particularly for salvage treatment of leukemias and lymphomas. In a 65-y-old woman with T-cell prolymphocytic leukemia and progressive disease after 4 lines of therapy, salvage treatment with [^90^Y]Y-Pentixather was offered, followed by autologous stem-cell transplantation. No further cancer drug therapy was performed. (A) Pretherapeutic [^68^Ga]Ga-Pentixafor PET/CT (3-dimensional view) identifies extensive radiotracer deposition in bilateral pulmonary manifestation sites and lymphatic/abdominal lesions. (B) Three-month follow-up [^68^Ga]Ga-Pentixafor PET/CT reveals significantly reduced tumor burden with local recurrence/progression of pleural and abdominal lesions (arrows).

The theranostic concept of CXCR4 targeting has moved swiftly from proof of principle (16) to clinical application. [^177^Lu]Lu-/[^90^Y]Y-Pentixather, the therapeutic counterpart of [^68^Ga]Ga-Pentixafor (11), currently represents the most advanced CXCR4-directed RPT in clinical use (30,31). Early-phase and compassionate-access studies consistently demonstrate substantial antitumor activity in hematologic malignancies (Fig. 2).

In acute myeloid and acute lymphoblastic leukemias, [^177^Lu]Lu-/[^90^Y]Y-Pentixather has primarily been used as a myeloablative conditioning regimen before hematopoietic stem-cell transplantation (25,32). Phase 1 and 2 trials have confirmed a favorable safety profile with dose escalation up to 10 GBq. Preliminary results indicate encouraging objective response rates, even in heavily pretreated or refractory patients. The ongoing PENTILULA trial (NCT06356922) will clarify the efficacy and long-term safety of this approach.

In multiple myeloma, CXCR4-directed therapy achieves high uptake and retention within myeloma lesions, correlating with strong response rates when combined with high-dose chemotherapy and stem-cell support (7,33). The COLPRIT trial (EudraCT 2015-001817-28) is investigating the integration of [^90^Y]Y-Pentixather into conditioning regimens to reduce or replace conventional high-dose chemotherapy.

Comparable results have been observed in relapsed or refractory lymphomas, where [^177^Lu]Lu-/[^90^Y]Y-Pentixather provides selective tumor targeting and measurable regression, including in aggressive T-cell and diffuse large B-cell subtypes (34). Early prospective data indicate prolonged progression-free survival and relevant reductions in tumor burden, with toxicity remaining predominantly hematologic and manageable. Beyond hematologic disease, CXCR4-targeted radionuclide therapy has shown promise in selected solid tumors characterized by high receptor expression. In desmoplastic small round cell tumors, early reports revealed measurable yet modest responses limited by bone marrow toxicity (35). Preclinical studies in SCLC using α-emitting [^212^Pb]Pb-Pentixather demonstrated significant delays in tumor growth and prolonged survival, suggesting that next-generation α-emitters may overcome the bone marrow tolerance constraints seen with β-emitters (36). Clinical translation of these findings into CXCR4-positive SCLC trials is under way (NCT05557708).

Across all tumor types, hematologic toxicity with cytopenia—neutropenia, thrombocytopenia, and anemia—remains the principal adverse event of CXCR4-targeted therapy (37). Grade 3 or greater cytopenias occur in roughly 70%–80% of patients. Hence, autologous or allogeneic hematopoietic stem-cell transplantation is mandatory. Notably, in hematologic malignancies, this myeloablative effect can be therapeutically desirable, potentially replacing the need for high-dose chemotherapy before transplantation (7,25). Renal toxicity is rare and usually related to tumor lysis or preexisting impairment, whereas hepatic effects are mild and transient.

Overall, CXCR4-targeted RPT achieves its greatest efficacy in hematologic malignancies, delivering robust disease control with a favorable safety profile when integrated into transplantation protocols. Early data in selected solid tumors suggest expanding opportunities. [^68^Ga]Ga-Pentixafor/[^177^Lu]Lu-/[^90^Y]Y-Pentixather–based theranostics exemplify the potential of molecularly guided theranostics, translating CXCR4 expression from a diagnostic biomarker into a therapeutic target and closing the loop between imaging and precision treatment.

## EXPANDING INDICATIONS BEYOND ONCOLOGY: CARDIOVASCULAR DISEASES

The CXCR4/CXCL12 axis also plays a crucial role in the recruitment of stem cells to damaged myocardium after myocardial infarction (MI); thus, chemokine receptor–directed PET has been used for patients with various cardiovascular diseases, including acute MI in a translational setting. Thackeray et al. were among the first to report an increased [^68^Ga]Ga-Pentixafor PET signal in a murine MI model up to 3 d after MI, followed by a rapid dissipation 4 d later (38). Flow cytometry provided evidence on a comparable reduction of leukocytes, whereas the PET signal at day 3 independently predicted the decline in myocardial function 6 wk after MI. Of note, animals treated using the maximum PET signal exhibited improved cardiac function relative to those treated using a dissipated PET signal, indicating that CXCR4-directed PET allows for image-guided treatment. Translational approaches in patients after MI highlighted a tight interaction between organs of systemic inflammatory response (spleen and bone marrow) and the infarct PET signal, which also correlated with ejection fraction at baseline. Of note, quantification of the in vivo PET signal in the infarct territory identified patients prone to later major cardiovascular events, thereby highlighting the predictive relevance of CXCR4-targeted molecular imaging after acute MI (39). Given its role in plaque instability, CXCR4-directed [^68^Ga]Ga-Pentixafor PET has also gained interest in other cardiovascular diseases, including atherosclerosis. Multiple studies have reported an increased PET signal in the calcified plaque burden of arterial wall segments, including in patients with cardiovascular risk factors. Application of theranostic counterparts of [^68^Ga]Ga-Pentixafor in hematologic diseases ([^177^Lu]Lu-/[^90^Y]Y-Pentixather) also revealed a potential antiinflammatory impact on atherosclerosis.

## ROLE OF CXCR4 THERANOSTICS IN OTHER INFLAMMATORY CONDITIONS

Beyond cardiovascular diseases, CXCR4-positive neutrophils have also been identified in COVID-19 infection, with chemokine receptor PET revealing multiple sites of inflammation, including distant sites of involvement not detected by conventional imaging (40). Preclinical investigations and an ongoing phase 2 study will determine the role of [^68^Ga]Ga-Pentixafor in giant cell arteritis (NCT05604482). Renal [^68^Ga]Ga-Pentixafor PET identified leukocyte infiltration in renal allograft infections (41), thereby expanding the clinical applications of chemokine receptor PET toward nephrology.

## CONCLUSION

CXCR4-targeted radiopharmaceuticals occupy a unique niche at the intersection of oncology, cardiology, immunology, and molecular imaging. Their promise lies in both diagnostic and therapeutic realms, particularly for hematologic malignancies, with potential extensions into solid tumors and nononcologic diseases.

However, despite the fact that imaging with [^68^Ga]Ga-Pentixafor has been in use for over a decade, a limited number of significant indications have been identified to date. In hematologic and oncologic diseases, this appears to be particularly evident in marginal zone lymphoma and multiple myeloma. The lack of results of prospective, randomized, multicenter trials makes it difficult to determine the reproducibility, clinical relevance, and cost-effectiveness of imaging CXCR4 expression. In the absence of this evidence, it remains uncertain whether CXCR4-targeted imaging can transition from a research tool to a clinical standard of care.

From a therapeutic standpoint, the use of CXCR4-targeted imaging in myeloma and certain lymphomas is of particular interest. In these settings, the target effect on tumor cells can be optimally exploited by an off-target effect (bone marrow aplasia before stem-cell transplantation). However, given the absence of any significant application of this approach in the context of other tumors, the therapeutic application of this approach remains limited by the presence of substantial side effects, usually necessitating stem-cell transplantation and the complexity of pretherapeutic dosimetry.

CXCR4-targeted radionuclide therapy using ^90^Y has been demonstrated to be a reliable modality for conditioning before stem-cell transplantation. This approach offers considerable potential in terms of obviating the necessity for high-dose chemotherapy in some patients, a development of particular interest for the treatment of children. This effect could potentially be intensified by the use of α-particles, such as ^225^Ac or ^211^At, in the future.

Finally, CXCR4 theranostics has been in use for over 10 y, demonstrating considerable potential. However, apart from a few niche indications, there has been no mass application to date, with the exception of primary aldosteronism (8). Thus, the clinical future of CXCR4 theranostics continues to face significant hurdles, including hematologic toxicity, biologic heterogeneity, and a lack of data from prospective studies.

## DISCLOSURE

PentixaPharm is the exclusive intellectual property license holder for Pentixafor, Pentixather (WO/2020/053256A1, EP/2019/074196W; WO/2015/185162A1, EP2014/061875; WO/2011/131735, EP2011/056358), and Pentixatec (EP21157225.0). This project received partial funding from the German Research Foundation (453989101 to Rudolf Werner, Takahiro Higuchi, and Andreas Buck). Margret Schottelius is listed as coinventor on the respective patents. Margret Schottelius and Andreas Buck are scientific advisors for PentixaPharm. Rudolf Werner and Andreas Buck have received speaker honoraria from Novartis/AAA and PentixaPharm and report advisory board work for Novartis/AAA and Bayer. No other potential conflict of interest relevant to this article was reported.
